# Prediction of post-acute care demand in medical and neurological inpatients: diagnostic assessment of the post-acute discharge score – a prospective cohort study

**DOI:** 10.1186/s12913-018-2897-0

**Published:** 2018-02-13

**Authors:** Antoinette Conca, Angela Gabele, Barbara Reutlinger, Philipp Schuetz, Alexander Kutz, Sebastian Haubitz, Lukas Faessler, Marcus Batschwaroff, Ursula Schild, Zeljka Caldara, Katharina Regez, Susanne Schirlo, Gabi Vossler, Timo Kahles, Krassen Nedeltchev, Anja Keller, Andreas Huber, Sabina De Geest, Ulrich Buergi, Petra Tobias, Martine Louis Simonet, Beat Mueller, Petra Schäfer-Keller

**Affiliations:** 1 Department of Clinical Nursing Science, Kantonsspital Aarau, Aarau, Switzerland; 2 University Department of Internal Medicine, Kantonsspital Aarau, Aarau, Switzerland; 3 Division of Infectious Diseases and Hospital Epidemiology, Kantonsspital Aarau, Aarau, Switzerland; 4 Department of Psychology, University of Berne, Berne, Switzerland; 5 Department for Neurology, Kantonsspital Aarau, Aarau, Switzerland; 6 Department of Social Services, Kantonsspital Aarau, Aarau, Switzerland; 7 Department of Laboratory Medicine, Kantonsspital Aarau, Aarau, Switzerland; 8 Institute of Nursing Science, University of Basel, Basel, Switzerland; 9 Emergency Department, Kantonsspital Aarau, Aarau, Switzerland; 10 Service of General Internal Medicine, University Hospitals Geneva, Geneva, Switzerland; 11 University of Applied Sciences and Arts Western Switzerland – School of Health Sciences Fribourg – HEdS-FR / HES-SO, Fribourg, Switzerland; 12 Pflege & MTTD, Fachabteilung Pflegeentwicklung. Kantonsspital Aarau, Tellstrasse 25, 5001 Aarau, CH Switzerland

**Keywords:** Discharge planning, Post-acute care discharge score, Diagnostic accuracy, Post-acute care facilities, Screening tool, Social worker referral

## Abstract

**Background:**

Early identification of patients requiring transfer to post-acute care (PAC) facilities shortens hospital stays. With a focus on interprofessional assessment of biopsychosocial risk, this study’s aim was to assess medical and neurological patients’ post-acute care discharge (PACD) scores on days 1 and 3 after hospital admission regarding diagnostic accuracy and effectiveness as an early screening tool. The transfer to PAC facilities served as the outcome (“gold standard”).

**Methods:**

In this prospective cohort study, registered at ClinicalTrial.gov (NCT01768494) on January 2013, 1432 medical and 464 neurological patients (total *n* = 1896) were included consecutively between February and October 2013. PACD scores and other relevant data were extracted from electronic records of patient admissions, hospital stays, and interviews at day 30 post-hospital admission. To gauge the scores’ accuracy, we plotted receiver operating characteristic (ROC) curves, calculated area under the curve (AUC), and determined sensitivity and specificity at various cut-off levels.

**Results:**

Medical patients’ day 1 and day 3 PACD scores accurately predicted discharge to PAC facilities, with respective discriminating powers (AUC) of 0.77 and 0.82. With a PACD cut-off of ≥8 points, day 1 and 3 sensitivities were respectively 72.6% and 83.6%, with respective specificities of 66.5% and 70.0%. Neurological patients’ scores showed lower accuracy both days: using the same cut-off, respective day 1 and day 3 AUCs were 0.68 and 0.78, sensitivities 41.4% and 68.7% and specificities 81.4% and 83.4%.

**Conclusion:**

PACD scores at days 1 and 3 accurately predicted transfer to PAC facilities, especially in medical patients on day 3. To confirm and refine these results, PACD scores’ value to guide discharge planning interventions and subsequent impact on hospital stay warrants further investigation.

**Trial registration:**

ClinialTrials.gov Identifier, NCT01768494.

**Electronic supplementary material:**

The online version of this article (10.1186/s12913-018-2897-0) contains supplementary material, which is available to authorized users.

## Background

Especially among geriatric patients, hospitalization with an acute medical condition is usually accompanied by reduced performance of activities of daily living (ADL) [1] and a range of other negative patient and economic outcomes [2, 3]. For example, during hospitalization, 35% of patients aged 70 years or older do not recover their preadmission status [1], resulting in high rates of transfer to post-acute care (PAC) facilities [2] and delays in hospital discharge. Mostly reflecting limited PAC housing capacity [4, 5] and lack of community support [6], and affecting mainly elderly, polymorbid and frail patients [1, 6], such delays increase the risk of mortality [2], nosocomial infection and the exacerbation of existing morbidities [3].

However, many of these delays and their concomitant losses, especially of functional ability, may be preventable via focused assessment and stratification of biopsychosocial risk, i.e., risk of requiring transfer to PAC facility, at or near admission [2, 7]. Along with assessment of in-patient care needs and functional deterioration, early measures should include initial post-discharge care planning [8–14] and timely involvement of social workers or case managers to plan transfers to PAC facilities.

While these actions will entail moderate administrative burdens, along with increased interprofessional teamwork and communication at admission, the potential reductions in stay lengths and improvements to patient outcomes support this exploration.

This paper focuses on interprofessional assessment of biopsychosocial risk. As possible measurement tools, we considered the Brass Index [15], the Self-Care Index (SPI; “Selbstpflegeindex”) [16] and the Social Work Admission Assessment Tool [8] all of which identify problems with inpatient discharge processes. However, none of these predict the need for PAC facility transfer.

In contrast, the Post-Acute Care Discharge (PACD) instrument is specifically designed to measure patients’ biopsychosocial risk and reliably predicts the need for transfers to PAC facilities [14]. PACD scores support interprofessional discussion in physician-nurse ward rounds [14] by identifying patients’ likelihoods of poor healthcare outcomes, informing actions and interventions to preserve functional status and arrange timely discharges. Therefore, for the current study, the PACD was selected as the most appropriate tool to identify the biopsychosocial risk of patients.

Therefore, the purpose of this study was to assess the prognostic accuracy of the PACD score at day 3 of hospital stay (PACD day-3) versus the PACD score at day 1 (PACD day-1) regarding transfer to a PAC facility in two distinct groups: medical and neurological inpatients.

## Methods

### Design and setting

This observational quality control study was embedded in a prospective cohort study conducted at the Cantonal Hospital Aarau (KSA; “*Kantonsspital Aarau*”). All details of the study protocol have been previously published [17]; the study is registered on the “ClinicalTrials.gov” (NCT01768494).

From February to October 2013, we included consecutive medical and neurological patients admitted to the KSA, which is a tertiary care hospital in Switzerland that also offers primary and secondary care services. On average, this hospital’s medical and neurological departments treat a combined total of 6000 inpatients per year. The Institutional Review Board of the Canton of Aargau approved the study and waived the need for informed consent (EK 2012/059) as this was an observational quality control study.

### Sample

We included consecutively admitted adult medical and neurological inpatients. We excluded those who were transferred to or from other hospitals, were admitted from PAC facilities, e.g., nursing homes, or died during the study period.

### Index testing

The current study applied two versions of the PACD: one administered within 24 h of admission (Additional file 1: Figure S1), and one for use on day 3 (Additional file 2: Figure S2). The first gathers data on fifteen variables: age, number of active medical problems on admission, ability of someone living with the patient to provide help at home, dependency in activities of daily living (7 ADL), and dependency in instrumental activities of daily living (5 IADL) during the last 2 weeks at home. The second calls for data on five variables: pre-admission medical problems, help provided at home, help with medication at home, dependency in bathing, and dependency regarding transfers from bed to chair on day 3 post-admission [14]. The original versions were developed on 349 patients admitted to general internal medicine wards, both PACD versions accurately predicted transfer to PAC facilities, with areas under the curve (AUCs) of 0.81 for the PACD day-1 and 0.82 for the PACD day-3 [14].

### Translation, scoring and validation of the PACDs

We translated the PACD instruments [14] from English to German conceptually and pilot tested them in a sample of 10 patients. Scoring principles for the PACD day-3 were developed by Louis Simonet et al. (2008). To allow referral of patients to social workers earlier than day 3, we transposed these principles to the PACD day-1 [14]. Points were attributed to each component based on the magnitudes of the day-1 model’s standardized regression coefficients in relation to one another, i.e., proportional point scores were assigned to each item [14]. We then analyzed as pre-tests the PACD scores for validity and feasibility in selected successive patient groups at the KSA. As measured by the PACD day-1 the biopsychosocial risk correlated significantly to discharge to a PAC facility, indicating predictive validity in the first evaluation of 240 patients with respiratory tract infections [18]. Based on this analysis, two adaptations were made. First, “transfer within the hospital” (part of the original PACD day-1 test) [14], was omitted because it was not significantly predictive of PAC facility transfer. Second, “partner to provide help,” was modified to "someone living with the patient to provide help" [9, 18]. The modified version was administered as second pre-test in our next sample of 308 patients who had suffered heart failure, urinary tract infections, falls, and syncope. Following our modifications, with a cut-off of ≥8, PACD day-1 scores showed a sensitivity of 91% and a specificity of 62% (AUC: 0.87). PACD day-3 scores showed a sensitivity of 82% and a specificity of 61% (AUC: 0.81) [19].

The scorings of the PACD day-1 and day-3 tests are shown in Additional file 1: Figure S1 and Additional file 2: Figure S2 [14]. The number of active medical problems, i.e., all current diagnoses of conditions with recognized therapeutic or diagnostic consequences, were scored as one point for each affected organ system (e.g., in patients with respiratory tract infection, two score points were calculated: one for a pulmonary condition and one for an infection) (Louis Simonet, personal communication on 17.05.2010).

The cut-off for both the day-1 and day-3 measurements was predefined as ≥8 points [19]. The PACD day-1 questions on patients’ pre-admission living situation and ADL/IADL were applicable to the context of Swiss emergency departments (ED) triage screening, i.e., the first evaluation of the PACD day-1 in patients with respiratory tract infections indicated its feasibility to assess patients in the ED setting [20].

The PACD scores were determined, applied, and included in patient records as part of discharge planning by physicians, nurses, and social workers. From their records, we extracted the data necessary to evaluate the predictive ability of the tool within the framework of this observational study. Given this method of data collection, the study could not be blinded.

### Outcome

Our two possible patient outcomes considered as “gold standard” were discharge to home and transfer to a PAC facility (i.e., nursing home, rehabilitation center, or other destination) [17].

### Data collection

Patients’ data were collected as part of routine clinical care from eligible neurological and medical patients admitted to hospital during the study period [17]. Treating physicians and nurses assessed the PACD day-1 scores in the ED. When PACD assessment was not possible in the ED, nurses assessed patients retrospectively in the medical ward. On the third day of the hospital stay, nurses assessed the PACD day-3 in the ward. Both scores were entered into the electronic patient record. The medical coding department collected data on pre-admission and post-discharge residence and length of stay from electronic patient records [17]. To assess post-discharge residence and other outcomes, specially trained study nurses contacted each patient 30 days after admission for a questionnaire-based telephone interview [17].

### Power calculation

To provide up to 40 degrees of freedom for our multivariable models, we aimed to include a total of 2000 patients over the course of 12 months, with an expected 20% rate of post-acute care facility transfers (*n* = 400) [17]. Power calculations for these models indicated that this sample size would have enough power to provide sufficient confidence intervals regarding the AUC, sensitivity, specificity, and positive and negative likelihood ratios (LRs), as well as for inter-group comparisons.

### Analysis

The patients’ characteristics were analyzed using means, standard deviations, medians, interquartile ranges, frequencies, and percentages, depending on scaling and distribution. To identify any unequal performance regarding the application of the PACD instruments, we separated the two patient groups for analysis. As recommended by Knottnerus et al. [21], PACD day-1 and 3 were analyzed using receiver operating characteristic (ROC) analysis to estimate the different cut-offs for sensitivity, specificity, positive and negative LRs, and the AUC. We stratified PACD risk groups into low (< 8), intermediate (8–15) and high risk (> 15) of requiring transfer to PAC facilities. For comparison between PACD patient groups, we used Mann-Whitney, Chi-squared and Kruskal-Wallis tests. Statistical analyses were performed using Stata IC 13 software.

A *p*-value of < 0.01 was considered significant, accounting for multiple testing.

## Results

**Fig. 1:**
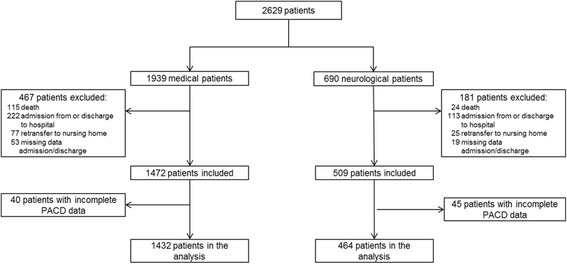
Flow chart of recruitment of medical and neurological patients (February – October 2013)

During the data collection period, 2629 patients were initially included in this study. Over the course of the data collection period, 733 (27.8%) were excluded for various reasons: death: 139 (5.3%); discharge to other hospital facilities: 335 (12.7%); re-transfer to nursing homes: 102 (3.9%); missing admission/discharge data: 72 (2.7%) and missing PACD scores: 85 (3.2%). The final test population consisted of 1896 subjects (medical patients: 1432; neurological patients: 464) (Fig.1). No significant differences regarding age, gender, number of active medical problems or self-care index (SPI) scores were found between test subjects and those without PACD data (Fig. 1).

### Patient characteristics

Medical patients had a mean age of 66 (±16) years, neurological patients 64 (±18) years. Both groups included slight majorities of men (medical: 58.7%; neurological: 57.3%). Most patients (medical: 89.4%, neurological: 78.7%) were discharged to their home. In total, 10.6% of medical and 21.3% of neurological patients were discharged to PAC facilities. In medical patients, 5.0% were transferred to nursing homes or other homes for the elderly and 5.6% to rehabilitation centers. In contrast, 20% of neurological patients were transferred to rehabilitation and only 1.3% to nursing homes or other homes for the elderly. Differences between patients discharged to PAC facilities or discharged to home after their hospital stays are shown in Tables 1 and 2. Patients discharged to PAC facilities registered higher dependence regarding ADLs and IADLs and this group’s mean length of hospital stay was more than double that of the group discharged to home. Detailed characteristics of medical and neurological patients are shown in Table 3.

**Table 1:** Characteristics of medical patients discharged to a PAC facility versus patients discharged home

Characteristics of the patients	discharged to a PAC facility	discharged home	*P* value
	*n* = 152	*n* = 1280	
Age: mean (SD); median [IQR]	75.3 (12.4); 77.5 [13.8]	65.1 (16.1); 68 [21]	<.001^a^
Number of men (%)	40.1	60.9	<.001^b^
PACD day-1: mean (SD); median [IQR]	11.4 (5.5); 10.5 [8]	6.5 (4.5); 5 [6]	<.001^a^
SPI first assessment on ward: mean (SD); median [IQR]	29.0 (7.5); 30 [10.0] *n* = 150	36.4 (5.3); 38 [5] *n* = 1192	<.001^a^
Number of active medical problems at admission: mean (SD); median [IQR]	4.0 (1.8); 4 [2]	3.1 (1.7); 3 [2]	<.001^a^
Number of self-reported disabilities in ADL and IADL: mean (SD); median [IQR]	3.7 (4.2); 2 [7] *n* = 147	1.2 (2.8); 0 [0] *n* = 1214	<.001^a^
Number of patients who live without someone able to provide help (%)	40.1	19.5	<.001^b^
Number of patients who need help in personal hygiene (%)	32.9	10.8	<.001^b^
Number of patients who need help in dressing/ undressing (%)	28.3	8.4	<.001^b^
Number of patients who need help in toileting (%)	14.5	5.7	<.001^b^
Number of patients who need help in bathing/ personal hygiene (%)	33.6	10.5	<.001^b^
Number of patients who need help in eating (%)	16.4	4.5	<.001^b^
Number of patients who need help in walking (%)	21.1	6.7	<.001^b^
Number of patients who need help in transfer (%)	17.8	4.8	<.001^b^
Number of patients who need help in travelling by car or public transportation (%)	40.1	13.7	<.001^b^
Number of patients who need help in shopping (%)	44.7	16.4	<.001^b^
Number of patients who need help in cooking (%)	38.8	13.8	<.001^b^
Number of patients who need help in homework (%)	46.7	18.0	<.001^b^
Number of patients who need help in medication (%)	32.2	10.9	<.001^b^
Length of hospital stay: mean (SD); median [IQR]	16.7 (12.3); 14 [11]	6.4 (6.2); 5 [5]	<.001^a^

^a^Mann-Whitney-U-Test

^b^Chi^2^-Test

**Table 2:** Characteristics of neurological patients discharged to a PAC facility versus patients discharged home

Characteristics of the patients	discharged to a PAC facility	discharged home	*P* value
	*n* = 99	*n* = 365	
Age: mean (SD); median [IQR]	71.2 (14.7); 76 [16]	62.0 (18); 66 [25]	<.001^a^
Number of men (%)	55.6	57.8	0.69^b^
PACD day-1: mean (SD); median [IQR]	6.8 (4.6); 6 [6]	4.3 (3.9); 3 [4]	<.001^a^
SPI first assessment on ward: mean (SD); median [IQR]	28.1 (9.1); 30 [17] *n* = 94	36.5 (5.3); 39 [5] *n* = 308	<.001^a^
Number of active medical problems at admission: mean (SD); median [IQR]	1.7 (0.8); 2 [1]	1.5 (1); 1 [1]	< 0.01^a^
Number of self-reported disabilities in ADL and IADL: mean (SD); median [IQR]	2.1 (3.7); 0 [5] *n* = 87	1 (2.7); 0 [0] *n* = 315	< 0.01^a^
Number of patients who live without someone able to provide help (%)	26.3	15.3	0.01^b^
Number of patients who need help in personal hygiene (%)	18.2	8.8	< 0.01^b^
Number of patients who need help in dressing/ undressing (%)	16.2	6.3	< 0.01^b^
Number of patients who need help in toileting (%)	12.1	6.3	0.05^b^
Number of patients who need help in bathing/ personal hygiene (%)	22.2	7.9	<.001^b^
Number of patients who need help in eating (%)	11.1	4.1	< 0.01^b^
Number of patients who need help in walking (%)	15.2	5.2	< 0.01^b^
Number of patients who need help in transfer (%)	16.2	4.9	<.001^b^
Number of patients who need help in travelling by car or public transportation (%)	24.2	11.5	< 0.01^b^
Number of patients who need help in shopping (%)	24.2	13.4	< 0.01^b^
Number of patients who need help in cooking (%)	22.2	10.4	< 0.01^b^
Number of patients who need help in homework (%)	26.3	12.3	< 0.01^b^
Number of patients who need help in medication (%)	20.2	8.8	< 0.01^b^
Length of hospital stay: median [IQR]	13.2 (5.4); 13 [5]	5.2 (4.5); 4 [3]	<.001^a^

^a^Mann-Whitney-U-Test

^b^Chi^2^-Test

**Table 3:** Characteristics of medical and neurological patients

Patient characteristics	medical patients	neurological patients
	*N* = 1432	*N* = 464
Age: mean (SD); median [IQR]	66.2 (16.0); 69 [21]	64.0 (17.7); 68 [24]
Number of men (%)	58.7	57.3
CD-10 main diagnosis (%)		
Infectious and parasitic diseases	13.3	3.4
Endocrine, nutritional and metabolic diseases	3.2	0.9
Diseases of the skin and subcutaneous tissue	0.9	–
Diseases of the respiratory system	12.2	0.2
Diseases of the eye and adnexa	–	1.1
Diseases of the blood and blood-forming organs and certain disorders involving the immune mechanism	1.9	0.2
Diseases of the circulatory system	27.7	36.4
Diseases of the musculoskeletal system and connective tissue	3.5	1.7
Diseases of the nervous system	0.7	39.4
Diseases of the ear and mastoid process	0.3	4.7
Diseases of the genitourinary system	3.8	0.4
Diseases of the digestive system	11.1	0.4
Neoplasms	10.1	1.7
Mental and behavioral disorders	2.0	2 .4
Pregnancy, childbirth and the puerperium	0.1	–
Symptoms, signs and abnormal clinical and laboratory findings	6.8	6.3
Injury, poisoning and certain other consequences of external causes	2.7	0.6
PACD day-1: mean (SD); median [IQR]	7.0 (4.8); 6 [7]	4.8 (4.2); 3.5 [5]
SPI^a^ first assessment ward: mean (SD); median [IQR];	35.6 (6.0); 38 [6] *n* = 1342	34.5 (7.3); 38 [8] *n* = 402
Active medical problems: mean (SD); median [IQR]	3.2 (1.7); 3 [2]	1.5 (1.0); 1 [1]
Discharge from the hospital to (%):		
Home	89.4	78.7
Nursing home^b^	4.0	1.1
Home for the elderly^b^	1.0	0.2
Rehabilitation^b^	5.6	20.0

^a^Self-care index

^b^PAC facilities

The PACD day-1 score was higher in medical patients (median: 6 [IQR: 7]) than in neurological patients (median: 3.5 [IQR: 5]).

### Diagnostic values of PACD day-1 and day-3 scores in medical and neurological patients

**Fig. 2:**
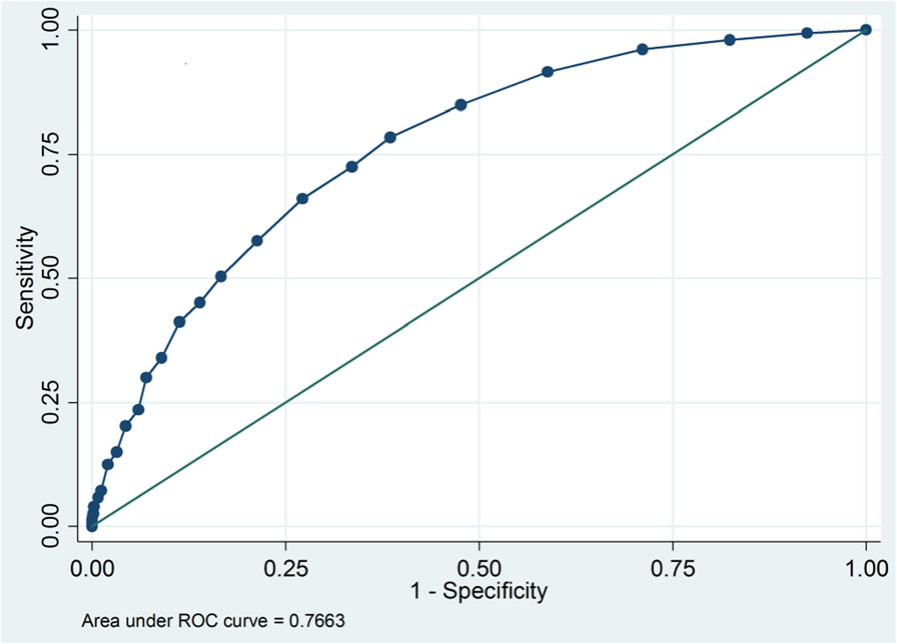
Receiver operator characteristic curve and AUC analysis of the PACD day-1 in medical patients

**Fig. 3:**
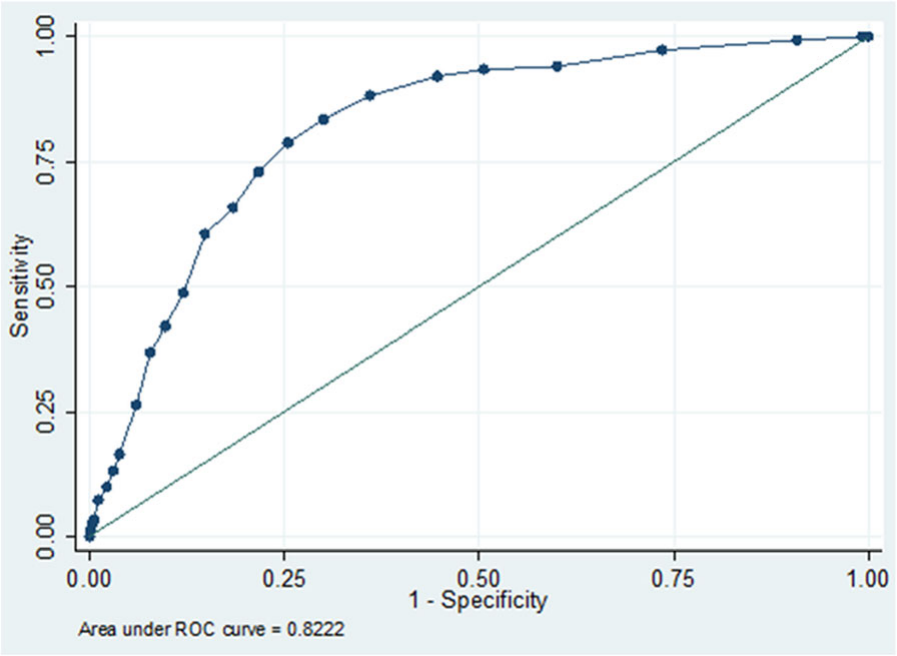
Receiver operator characteristic curve and AUC analysis of PACD day-3 in medical patients

The medical patients’ PACD day-1 data yielded an AUC of 0.77. At the pre-specified cut-off ≥8 points, sensitivity was 72.6% and specificity was 66.5% (Fig. 2). Lowering the cut-off to ≥7 points resulted in a sensitivity of 78.4% and a specificity of 61.5%. For this group’s PACD day-3 data, using the cutoff of ≥8 points, the AUC was 0.82, sensitivity 83.6% and specificity 70.0% (Fig. 3).

**Fig. 4:**
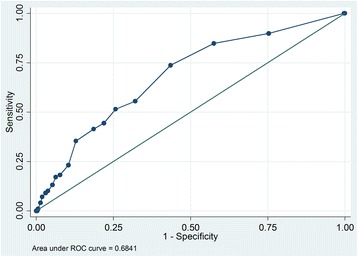
Receiver operator characteristic curve and AUC analysis of the adapted PACD day-1 in neurological patients

**Fig. 5:**
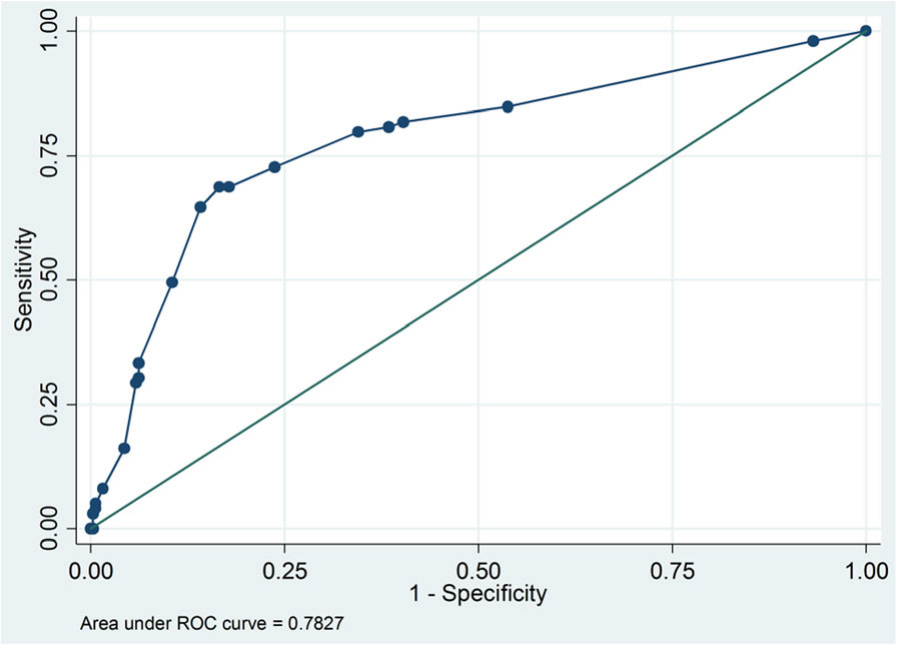
Receiver operator characteristic curve and AUC analysis of PACD day-3 in neurological patients

In neurological patients, again using a cut-off of ≥ 8 points, the PACD day-1 AUC was 0.68, with a sensitivity of 41.4% and specificity of 81.4% (Fig. 4). Lowering the cut-off to ≥6 increased the sensitivity to 51.2% and decreased the specificity to 74.3%. For PACD day-3, with a ≥ 8 point cutoff, the AUC increased from the corresponding day-1 level to 0.78, with 68.7% sensitivity and 83.4% specificity (Fig. 5).

Additional cut-offs for PACD day-1 and day-3 and the corresponding sensitivities, specificities, positive and negative likelihood ratios, including confidence intervals are documented in Additional file 3: Tables S1-S4.

### Length of stay, discharge destination and age in PACD risk groups

With a mean length of stay of 10 days, patients whose PACD day-1 or day-3 scores indicated intermediate (i.e., PACD = 8–15) or high (i.e., PACD > 15) risk had 67% longer hospital stays than those whose scores indicated a low (PACD < 8) risk (mean length of stay: 6 days). This proportion was similar in both the medical and the neurological group. Medical patients screened by the PACD day-3 showed the greatest range in lengths of stay: on average, low-risk patients stayed 6 days, intermediate-risk patients stayed 9 days and high-risk patients stayed 14 days. Across the entire sample, patients identified as low-risk had the lowest rates of transfer to PAC facilities, with 0.7%, 0.2%, 7.7% discharged respectively to nursing homes, rehabilitation centers, or intermediate elder care homes. In the intermediate group, these figures were 4.4%, 3.2%, and 21.4%, respectively; the high-risk group’s respective admission rates were 13.9%, 4.8%, and 35.4%. On average, patients whose PACD day-1 or day-3 data indicated intermediate or high risk were older than those with low risk, both in the medical (75 vs. 61 years) and the neurological (74 vs. 60 years) group (Table 4).

**Table 4:** Length of stay and living situation after discharge for PACD risk groups

	Medical patients						Neurological patients					
	PACD day-1 (n = 1432)			PACD day-3 (*n* = 1325)			PACD day-1 (n = 464)			PACD day-3 (*n* = 423)		
Risk groups^a^	< 8	8–15	> 15	< 8	8–15	> 15	< 8	8–15	> 15	< 8	8–15	> 15
Number of patients (n)	892	428	112	555	625	145	355	95	14	256	147	20
Length of stay (mean)^*^	6.3	9.3	9.6	5.7	8.7	13.8	6.1	9.5	9.4	5.7	9.6	12.7
Age (mean)^*^	60.6	74.5	79.4	61.5	70.9	73.9	60.5	76.0	72.3	60.1	69.9	76.0
Living situation after hospital discharge: %												
At home	95.3	82.5	68.8	97.3	87.8	58.6	83.7	64.2	50.0	90.6	59.2	25.0
Nursing home	1.0	7.2	15.2	0.7	3.8	19.3	0.3	3.2	7.1	0.0	3.4	0.0
Home for the elderly	0.1	1.9	5.4	0.2	1.3	4.1	0.3	0.0	0.0	0.0	0.0	5.0
Rehabilitation	3.6	8.4	10.7	1.8	7.0	17.9	15.8	32.6	42.9	9.4	37.4	70.0

^a^low risk (< 8), intermediate risk (8–15), high risk (> 15)

^*^p < .01, Kruskal Wallis-Test

## Discussion

This large-scale study in medical and neurological patients assessed the diagnostic accuracy of the original PACD day-3 instrument versus that adapted for day-1 use, and analyzed the predictive value of each regarding PAC facility transfer. Particularly in medical patients, both day-1 and day-3 scores had remarkable predictive accuracy in determining patients’ risks of requiring PAC transfer. For screening purposes in medical patients, the PACD day-1 and day-3 data yielded good AUCs and sensitivity.

Compared to the AUC of the previously used day-3 model [14] the results were similar for our day-3 model (each AUC = 0.82); however, for our day-1 model results differed. While the AUC for medical patients (AUC: 0.77) fell within the confidence interval of the previously used model (AUC: 0.81, 95% CI 0.76–0.86), that for neurological patients (AUC: 0.66) was lower. While our day-3 model can be directly compared to its forerunner (given the identical scoring of the two), our day-1 model cannot. Our model had one fewer item, and was tested in a more aged sample (mean age 75.3y versus 71.0y) with more active medical problems on admission day (4.0 vs. 2.1) and a smaller proportion of males (40.1% vs. 47.0%). Perhaps most importantly, the score was measured and implemented under actual clinical conditions.

Interestingly, higher age per se and greater numbers of medical problems may not necessarily translate into more discriminant power concerning discharge to a PAC facility. In fact, while these data are readily available and thus possibly less expensive and simpler to implement than PACD screening, our data do not support the use of patient age to define risk groups in relation to PAC needs. Sensitivity analyses yielded AUC ranging from 0.68–0.72 in stratified groups aged ≥80 – ≥60 for the day-1 model and 0.57–0.67 in similarly stratified groups for the day-3 model in medical patients. In this model, AUC (data not shown) were even lower for neurological patients. Moreover, an age stratification approach would have failed to detect PAC facility need for 14 patients aged < 60 years, 38 patients aged < 70 years, and 94 patients aged < 80 years in our day-1 model (similar numbers in day-3 model, data not shown), a number which we consider noteworthy.

Further, concerning the less compelling results in neurological patients compared to those in medical patients, for these patients–many of whom were admitted for stroke–“discharge to a PAC facility” was likely driven more by newly acquired functional deficits than by those tested by the PACD day-1 instrument, i.e., manifesting over the two weeks prior to admission. In neurological patients, then, the PACD score was accurate for risk determination on admission day in its current form. However, PACD day 3 with a cut-off of ≥8 produced an AUC of 0.78, making it a promising screening tool in this group.

Our results for PACD day-3 scores in medical patients (AUC: 0.82; sensitivity: 84%; specificity: 70%) are in line with the findings of Louis Simonet et al. (AUC: 0.82; sensitivity: 87%; specificity: 63%) [14]. Also supporting Louis Simonet et al. regarding the discriminatory power of PACD scores ≥8 on day 3 in a clinical setting, scores for patients with heart failure, urinary tract infections, falls, or syncope registered the highest sensitivity: 91%, with a specificity of 62% [19]. Although this study was conducted in a Swiss setting with a moderate to high access to PAC services, the PACD could still be applied in settings with lower PAC availability. The earlier patients’ biopsychosocial risk can be identified, the more they can benefit from tailored discharge preparation.

We used a single cut-off to define patient risk. For clinical decision-making (i.e., to optimize length of stay), i.e., to prioritize the patients most likely to need social workers or case managers, differentiating medium- from high-risk cases might be preferable to a simple PAC/no PAC dichotomy.

Despite the better AUC and sensitivity of PACD day-3 data, with a cut-off ≥8, the PACD day-1 has the advantage of informing discharge planning from the earliest possible moment after admission. The main goals of early screening are to minimize waiting times for transfer to appropriate PAC facilities, to optimize patient functional status during hospitalization, and to optimize preparation for discharge. A lack of PAC facility vacancies may increase LOS. We experienced this in our own previous work, where it led to an accumulated waiting time of 220 days in 61 patients (unpublished data (Albrich et al., 2013), reported by others as the main reason for nonmedical delays, accounting for between 40% (Selker et al. 1989) and 84% (Carey 2005) of total delayed days). We therefore propose that, within 24 h of admission, the clinical team could compile a list of at-risk patients (PACD ≥8), who could then be screened by the social workers themselves, maximizing the time available to find appropriate solutions. As levels both of impairment in (instrumental) activities of daily living and of the availability of assistance at home are valuable information for discharge planning, we estimate that collecting the remaining PACD-specific information will require minimal additional effort. To that end, we recommend that the PACD to be integrated into a bundle of discharge-optimizing interventions. While this would admittedly require increased staff resources at admission, the cost would be offset by reductions in length of stay.

### Potential limitations and risk of bias

It was impossible to blind the PACD to clinicians as they used it in clinical practice. Therefore the PACD could have been used to prioritize social worker involvement in post-acute care planning, i.e., higher risk patients may have been preferentially admitted to PAC. However, we found no indications of this in our 30-day follow-up interviews. Moreover, Louis Simonet et al.’s previous study (2008) largely supports our findings on the PACD scores’ diagnostic value regarding discharge to PAC facilities [14].

## Conclusion

PACD scores at days 1 and 3 accurately predict transfer to a PAC facility, especially in medical patients. Through early identification of patients’ care needs (part of the PACD’s function), especially the need for later transfer to PAC facilities, application of the PACD day-1 and day-3 instruments can reduce the risk of hospital-acquired disability and length of stay. By aiding rational allocation of limited healthcare resources, we consider this study to be highly relevant to the Swiss healthcare system. To determine whether improved patient triage via PACD translates into more efficient management and improved patient outcomes, an intervention study is needed.

## Additional files

Additional file 1: Figure S1. Scoring of the PACD day-1. (JPEG 664 kb)

Additional file 2: Figure S2. Scoring of the PACD day-3. (JPEG 685 kb)

Additional file 3: Tables S1-S4. Illustrating the sensitivity, specificity and AUC (CI: 95%) values for PACD day 1 and PACD day 3 in medical and in neurological patients. (DOCX 23 kb)

## Abbreviations

ADL, IADL: Activities of daily living, instrumental activities of daily living
AUC: Area under the curve
CI: Confidence interval
ED: Emergency department
KSA: Cantonal Hospital Aarau
LR+/LR-: Likelihood ratio positive, negative
PAC: Post-acute care
PACD: Post-acute care discharge
SD: Standard deviation

## References

[1] Boyd CM, Landefeld CS, Counsell SR, Palmer RM, Fortinsky RH, Kresevic D, Burant C, Covinsky KE (2008). Recovery of activities of daily living in older adults after hospitalization for acute medical illness. J Am Geriatr Soc.

[2] McMartin K (2013). Discharge planning in chronic conditions: an evidence-based analysis. Ontario health technology assessment series.

[3] Rosman M, Rachminov O, Segal O, Segal G (2015). Prolonged patients' in-hospital waiting period after discharge eligibility is associated with increased risk of infection, morbidity and mortality: a retrospective cohort analysis. BMC Health Serv Res.

[4] Lenzi J, Mongardi M, Rucci P, Di Ruscio E, Vizioli M, Randazzo C, Toschi E, Carradori T, Fantini MP (2014). Sociodemographic, clinical and organisational factors associated with delayed hospital discharges: a cross-sectional study. BMC Health Serv Res.

[5] McDonagh MS, Smith DH, Goddard M (2000). Measuring appropriate use of acute beds. A systematic review of methods and results. Health policy (Amsterdam, Netherlands).

[6] Tran B, Zureik M, Davido A, Levy A, Trouillet JL, Lang T, Lombrail P (1995). Hospital discharge planning and length of hospital stay in elderly patients admitted through the emergency department. Revue d'epidemiologie et de sante publique.

[7] Fox MT, Persaud M, Maimets I, Brooks D, O'Brien K, Tregunno D (2013). Effectiveness of early discharge planning in acutely ill or injured hospitalized older adults: a systematic review and meta-analysis. BMC Geriatr.

[8] Boutin-Foster C, Euster S, Rolon Y, Motal A, BeLue R, Kline R, Charlson ME (2005). Social work admission assessment tool for identifying patients in need of comprehensive social work evaluation. Health & social work.

[9] Albrich WC, Ruegger K, Dusemund F, Bossart R, Regez K, Schild U, Conca A, Schuetz P, Sigrist T, Huber A (2011). Optimised patient transfer using an innovative multidisciplinary assessment in Kanton Aargau (OPTIMA I): an observational survey in lower respiratory tract infections. Swiss Med Wkly.

[10] Albrich WC, Ruegger K, Dusemund F, Schuetz P, Arici B, Litke A, Blum CA, Bossart R, Regez K, Schild U (2013). Biomarker-enhanced triage in respiratory infections: a proof-of-concept feasibility trial. Eur Respir J.

[11] Dusemund F, Steiner M, Vuilliomenet A, Muller C, Bossart R, Regez K, Schild U, Conca A, Huber A, Reutlinger B (2012). Multidisciplinary assessment to personalize length of stay in acute decompensated heart failure (OPTIMA II ADHF). J Clin Med Res.

[12] Holland DE, Mistiaen P, Bowles KH (2011). Problems and unmet needs of patients discharged "home to self-care". Prof Case Manag.

[13] Bowles KH, Ratcliffe SJ, Holmes JH, Liberatore M, Nydick R, Naylor MD (2008). Post-acute referral decisions made by multidisciplinary experts compared to hospital clinicians and the patients' 12-week outcomes. Med Care.

[14] Louis Simonet M, Kossovsky MP, Chopard P, Sigaud P, Perneger TV, Gaspoz JM (2008). A predictive score to identify hospitalized patients' risk of discharge to a post-acute care facility. BMC Health Serv Res.

[15] Mistiaen P, Duijnhouwer E, Prins-Hoekstra A, Ros W, Blaylock A (1999). Predictive validity of the BRASS index in screening patients with post-discharge problems. Blaylock risk assessment screening score. J Adv Nurs.

[16] große Schlarmann J: Der CMS© im ePA©. Verschiedene Qualitätsdimensionen eines Instruments. Eine empirische Analyse. Gelsenkirchen: Private Universität Witten/Herdecke gGmbH; 2007.

[17] Schuetz P, Hausfater P, Amin D, Haubitz S, Fassler L, Grolimund E, Kutz A, Schild U, Caldara Z, Regez K (2013). Optimizing triage and hospitalization in adult general medical emergency patients: the triage project. BMC Emerg Med.

[18] Conca A, Bossart R, Regez K, Schild U, Wallimann G, Schweingruber R, Tobias P, Albrich WC, Ruegger K (2012). Dusemund F *et al*: [OPTIMA - optimized patient transfer through innovative multidisciplinary assessment: project description phase I]. Pflegewissenschaft.

[19] Conca A, Gabele A, Regez K, Brunner C, Schild U, Guglielmetti M, Sebastian S, Schäfer-, Keller P, Schirlo S *et al*: Erfassung eines Nachakutpflegebedarf bei hospitalisierten, medizinischen Patienten durch die „Post-Acute Care Discharge scores“ (PACD) *Pflegewissenschaft* 2015(11):584–597.

[20] Conca A, Regez K, Schild U, Reutlinger B, Schafer P, Schweingruber R, Tobias P, Burgi U, Schirlo S, Muller B (2013). At admission planning discharge already. Krankenpfl Soins Infirm.

[21] Knottnerus JA, van Weel C, Muris JW (2002). Evaluation of diagnostic procedures. BMJ.

