# Supplementary figures and images for: Prediction of post-acute care demand in medical and neurological inpatients: diagnostic assessment of the post-acute discharge score – a prospective cohort study
Source: BMC Health Serv Res. 2018 Feb 13;18:111. doi: 10.1186/s12913-018-2897-0 (PMC5812184; doi:10.1186/s12913-018-2897-0)

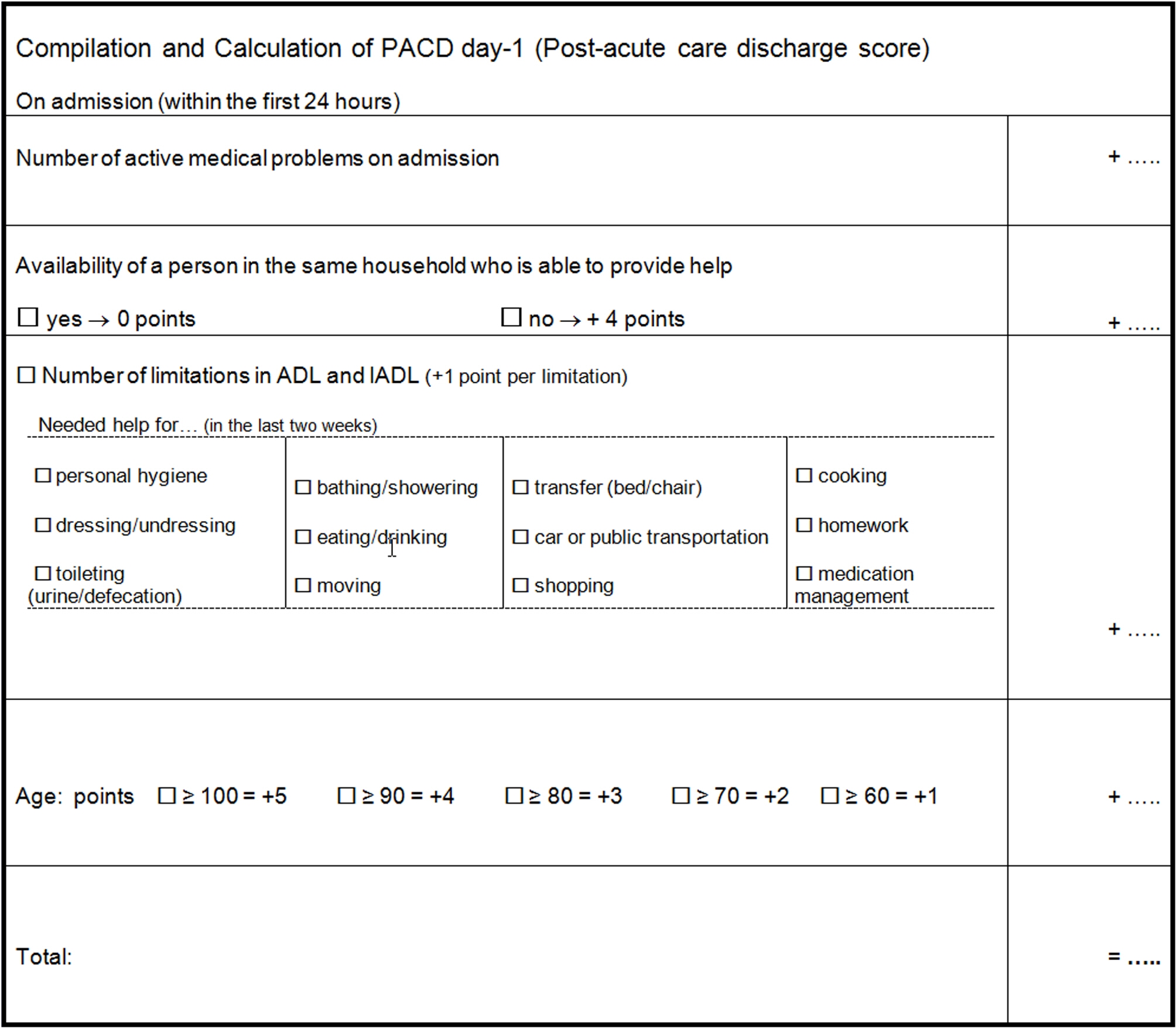

Supplement: Supplementary file 1 — Scoring of the PACD day-1. (JPEG 664 kb) [file 12913_2018_2897_MOESM1_ESM.jpg]

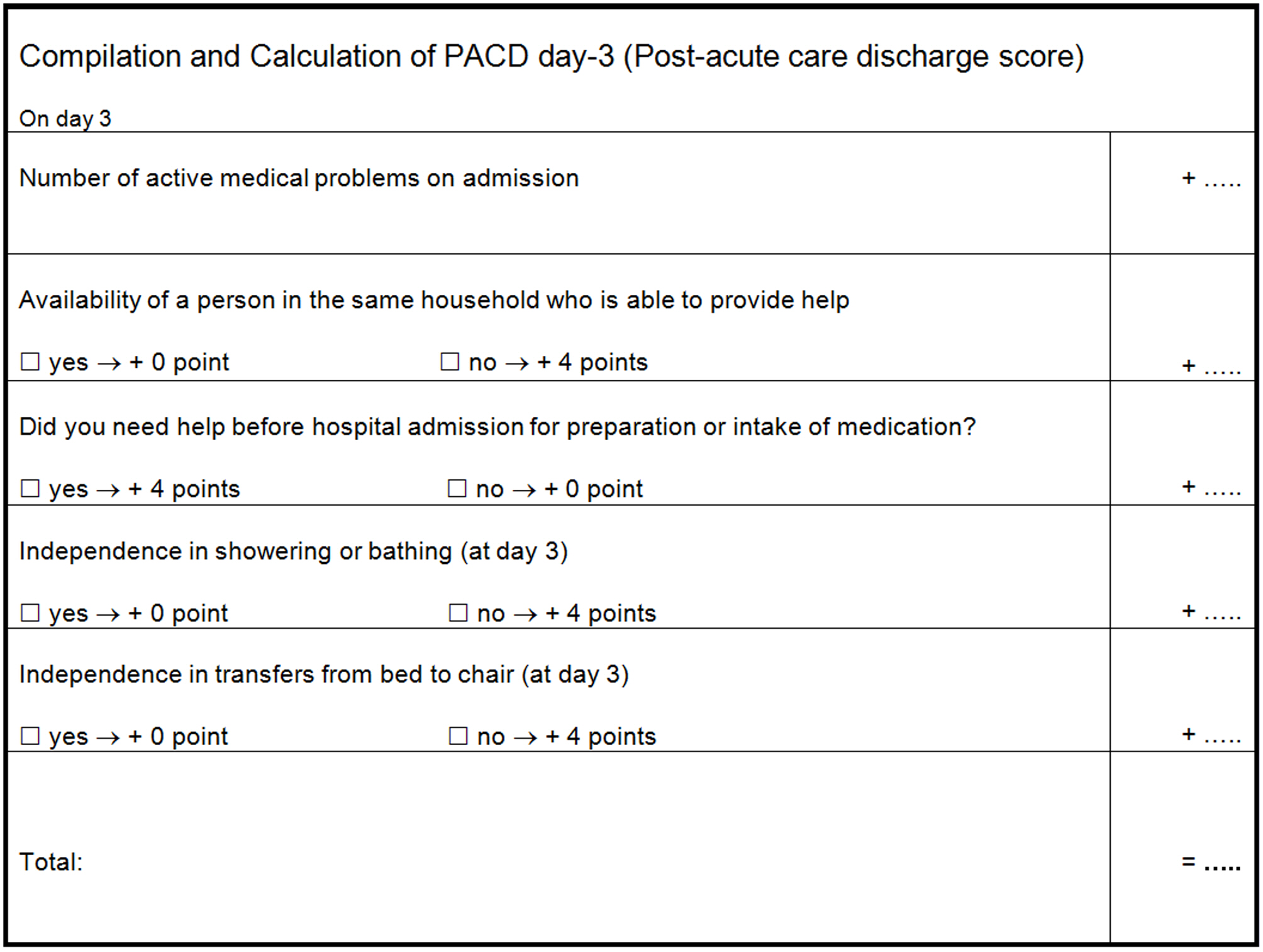

Supplement: Supplementary file 2 — Scoring of the PACD day-3. (JPEG 685 kb) [file 12913_2018_2897_MOESM2_ESM.jpg]
