# Supplementary material for: Prediction of post-acute care demand in medical and neurological inpatients: diagnostic assessment of the post-acute discharge score – a prospective cohort study
Source: BMC Health Serv Res. 2018 Feb 13;18:111. doi: 10.1186/s12913-018-2897-0 (PMC5812184; doi:10.1186/s12913-018-2897-0)
Supplement: Supplementary file 3 — Illustrating the sensitivity, specificity and AUC (CI: 95%) values for PACD day 1 and PACD day 3 in medical and in neurological patients. (DOCX 23 kb) [file 12913_2018_2897_MOESM3_ESM.docx]

**Additional files**

| **Cut-off** | **Sensitivity (CI 95%)** | **Specificity (CI 95%)** | **LR +**  **(CI 95%)** | **LR-**  **(CI 95%)** |
| --- | --- | --- | --- | --- |
| ≥ 6 | 85.0%  (78.1% to 90.0%) | 52.4% (49.6% to 55.2%) | 1.8 (1.6 to 2.0) | 0.3 (0.2 to 0.4) |
| ≥ 7 | 78.4% (70.9% to 84.5%) | 61.5% (58.7% to 64.1%) | 2.0 (1.8 to 2.3) | 0.4  (0.3 to 0.5) |
| ≥ 8 | 72.6% (64.6% to 79.3%) | 66.5% (63.8% to 69.0%) | 2.2 (1.9 to 2.5) | 0.4 (0.3 to 0.5) |
| ≥ 9 | 66.0% (57.9% to 73.4%) | 72.9% (70.3% to 75.3%) | 2.4 (2.1 to 2.8) | 0.5 (0.4 to 0.6) |
| ≥ 10 | 57.5% (49.3% to 65.4%) | 78.7% (76.3% to 80.9%) | 2.7 (2.3 to 3.2) | 0.5 (0.5 to 0.7) |
| AUC: 0.77 | Std.Err: 0.02 | CI (95%): 0.73 to 0.80 | | |

Table S1: Sensitivity, specificity and AUC (CI: 95%) of the adapted PACD day-1 in medical patients

| **Cut-off** | **Sensitivity (CI 95%)** | **Specificity (CI 95%)** | **LR +**  **(CI 95%)** | **LR-**  **(CI 95%)** |
| --- | --- | --- | --- | --- |
| ≥ 6 | 92.1% (86.3% to 95.7%) | 55.4% (52.5% to 58.3%) | 2.1 (1.9 to 2.2) | 0.1 (0.1 to 0.3) |
| ≥ 7 | 88.2% (81.7% to 92.6%) | 64.0% (61.2% to 66.7%) | 2.5  (2.2 to 2.7) | 0.2 (0.1 to 0.3) |
| ≥ 8 | 83.6% (76.5% to 88.9%) | 70.0%  (67.2% to 72.6%) | 2.8  (2.5 to 3.1) | 0.2 (0.2 to 0.3) |
| ≥ 9 | 79.0% (71.4% to 85.0%) | 74.6% (72.0% to 77.0%) | 3.1 (2.7 to 3.5) | 0.3 (0.2 to 0.4) |
| ≥ 10 | 73.0% (65.1% to 79.8%) | 78.4% (75.9% to 80.7) | 3.4 (2.9 to 3.9) | 0.3 (0.3 to 0.5) |
| AUC: 0.82 | Std.Err: 0.02 | CI (95%): 79.0% to 85.5% | | |

Table S2: Sensitivity, specificity and AUC (CI: 95%) of the adapted PACD day-3 in medical patients

| **Cut-off** | **Sensitivity (CI 95%)** | **Specificity (CI 95%)** | **LR +**  **(CI 95%)** | **LR-**  **(CI 95%)** |
| --- | --- | --- | --- | --- |
| ≥ 6 | 51.5% (41.3% to 61.6%) | 74.3% (69.7% to 78.6%) | 2.0  (1.5 to 2.6) | 0.7 (0.5 to 0.8) |
| ≥ 7 | 44.4% (34.6% to 54.8%) | 78.1% (73.4% to 82.2%) | 2.0  (1.5 to 2.7) | 0.7 (0.6 to 0.9) |
| ≥ 8 | 41.4% (31.7% to 51.8%) | 81.4% (76.9% to 85.2%) | 2.2 (1.6 to 3.1) | 0.7 (0.6 to 0.9) |
| ≥ 9 | 35.4% (26.2% to 45.7%) | 87.1% (83.2% to 90.3%) | 2.8 (1.9 to 4.0) | 0.7 (0.6 to 0.9) |
| ≥ 10 | 23.2% (15.6% to 33.0%) | 89.6% (85.9% to 92.4%) | 2.2 (1.4 to 3.6) | 0.9 (0.8 to 1.0) |
| AUC: 0.68 | Std.Err: 0.03 | CI (95%): 62.6% to 74.2% | | |

Table S3: Sensitivity, specificity and AUC with (CI: 95%) of the adapted PACD day-1 in neurological patients

| **Cut-off** | **Sensitivity (CI 95%)** | **Specificity (CI 95%)** | **LR +**  **(CI 95%)** | **LR-**  **(CI 95%)** |
| --- | --- | --- | --- | --- |
| ≥ 6 | 72.7% (62.7% to 81.0%) | 76.3% (71.2% to 80.8%) | 3.1 (2.4 to 3.9) | 0.4 (0.3 to 0.5) |
| ≥ 7 | 68.7% (58.5% to 77.4%) | 82.2% (77.5% to 86.1%) | 3.9 (2.9 to 5.0) | 0.4 (0.3 to 0.5) |
| ≥ 8 | 68.7% (58.5% to 77.4%) | 83.4% (78.8 to 87.2%) | 4.1 (3.1 to 5.5) | 0.4 (0.3 to 0.5) |
| ≥ 9 | 64.7% (54.3% to 73.8%) | 85.9% (81.5% to 89.4%) | 4.6 (3.4 to 6.2) | 0.4 (0.3 to 0.5) |
| ≥ 10 | 49.5% (39.4% to 59.7%) | 89.6% (85.6% to 92.6%) | 4.7 (3.3 to 6.9) | 0.6 (0.5 to 0.7) |
| AUC: 0.78 | Std.Err: 0.03 | CI (95%): 72.7% to 83.8% | | |

Table S4: Sensitivity, specificity and AUC with CI (95%) of the adapted PACD day-3 in neurological patients
